# Gut Microbiota and Dietary Intake Across Training Phases in Competitive Long-Distance Runners: An Exploratory Longitudinal Study

**DOI:** 10.3390/nu18183036

**Published:** 2026-09-17

**Authors:** Chun-Yu Kuo, Wan-Chun Chiu, Ting-Yin Chou, Yi-Ju Hsu

**Affiliations:** 1 Graduate Institute of Sports Science, National Taiwan Sport University, Taoyuan City 33301, Taiwan; 1131301@ntsu.edu.tw; 2 School of Nutrition and Health Sciences, College of Nutrition, Taipei Medical University, Taipei City 11031, Taiwan; wanchun@tmu.edu.tw; 3 Department of Sports Training Science-Athletics, National Taiwan Sport University, Taoyuan City 33301, Taiwan; alan5571@ntsu.edu.tw

**Keywords:** nutritional intake, gut microbiota, endurance athletes, energy intake, training phase

## Abstract

**Background/Objectives**: In this exploratory longitudinal study, we examined dietary intake and gut microbiota across preparation, competition, and transition phases in competitive long-distance runners and explored microbial patterns associated with phase-specific dietary intake. **Methods**: Seven competitive long-distance runners were assessed across three training phases. At each phase, dietary intake and body composition were assessed, and fecal samples were collected. Gut microbiota composition was analyzed using 16S rRNA gene sequencing. Phase-related taxonomic differences were evaluated using repeated-measures approaches with multiple-testing correction, and differential abundance analysis was performed using DESeq2 with participant identity included as a blocking factor. Additionally, exploratory k-means clustering based on energy, carbohydrate, and protein intake was used to characterize dietary intake patterns. **Results**: Energy, protein, and fat intake differed significantly across training phases, whereas carbohydrate intake did not reach statistical significance. Exploratory alpha- and beta-diversity analyses yielded no statistically detectable differences across phases. At the phylum level, *Synergistetes* showed an overall phase effect after FDR correction, although no pairwise comparison remained significant after Bonferroni adjustment. In the DESeq2 analysis, *Haemophilus* was more abundant during the transition than preparation phase, *Enterococcus* was more abundant during preparation than transition, and *Cronobacter* was more abundant during preparation than competition (all adjusted *p* < 0.05). Exploratory dietary clustering identified additional cluster-associated microbial differences, but cluster membership substantially overlapped with the training phase. **Conclusions**: Dietary intake varied across the competitive training cycle. Community-level diversity results were presented for exploratory purposes only in this small cohort. A limited number of taxa showed phase-related differences after multiple-testing correction. Because dietary intake and training phase changed concurrently, the cluster-associated microbial findings should be interpreted as exploratory patterns within the combined nutritional and training context rather than as independent dietary effects.

## 1. Introduction

The gut microbiota represents a highly dynamic and metabolically active ecosystem that plays a crucial role in maintaining host homeostasis, regulating energy metabolism, and modulating immune and inflammatory responses [1,2]. Microbial metabolites, particularly short-chain fatty acids (SCFAs), have been suggested to influence exercise performance through their roles in mitochondrial function, substrate utilization, and metabolic regulation [3,4]. In endurance athletes, sustained oxidative metabolism and high carbohydrate turnover may shape gut microbial functions relevant to substrate utilization, gastrointestinal tolerance, and recovery [5,6]. Consistent with this, endurance training has been associated with the enrichment of SCFA-producing taxa and maintenance of mucin-degrading species linked to metabolic efficiency and intestinal integrity [7]. Experimental evidence further demonstrates that *Veillonella atypica*, identified in endurance athletes, can utilize exercise-derived metabolites to produce compounds that may influence endurance performance [8].

Dietary intake remains one of the most influential modulators of the gut microbiota [9,10]. In endurance athletes, energy and macronutrient intake vary across distinct training phases, including preparation, competition, and transition, reflecting differences in training demands and performance goals [11,12]. During the preparation phase, athletes often manipulate carbohydrate availability to promote metabolic adaptations, including enhanced mitochondrial function and substrate utilization [11]. In contrast, during the competition phase, athletes typically adopt a higher carbohydrate intake to maintain glycogen availability, support high-intensity performance, and reduce fatigue [13,14].

Despite growing interest in the gut microbiome of athletes, important knowledge gaps remain regarding how microbial communities respond to the dynamic nutritional and training environments encountered during periodized endurance training. Although previous studies and reviews have highlighted this area, much of the existing evidence remains cross-sectional, and findings in endurance athletes have been inconsistent, potentially reflecting differences in dietary intake, training demands, and sampling timing relative to the competition phase [7,15].

Some longitudinal studies have reported limited changes in broad community-level diversity measures alongside variation in specific microbial taxa [16,17]. However, how gut microbial composition and diversity vary across different phases of a competitive training cycle in long-distance runners remains insufficiently characterized. Therefore, the aim of this study was to longitudinally characterize gut microbiota composition and diversity across preparation, competition, and transition phases and explore how observed microbial patterns correspond with phase-specific dietary intake. Exploratory dietary clustering was additionally applied to characterize microbial patterns accompanying higher- and lower-intake profiles across phase comparisons. We hypothesized that community-level diversity would show limited phase-related variation, whereas selected taxa previously associated with endurance exercise, particularly SCFA-producing and lactate-utilizing taxa, such as *Veillonella*, might vary across the training cycle.

## 2. Materials and Methods

### 2.1. Participants

A total of 7 healthy competitive long-distance runners (5 males and 2 females) were recruited from a university track and field team. Their training experience ranged from 7 to 16 years. These seven athletes represented all eligible participants who were enrolled and completed the study and were not selected as a subset from a larger enrolled sample. Inclusion criteria required that participants had not consumed any lactic acid bacteria-related foods or probiotics during the three months preceding the study and had no history of food allergies, cardiovascular diseases, hypertension, metabolic disorders, or asthma. Participants were excluded if they had experienced neuromuscular injuries within the previous six months. All participants provided written informed consent before participation. The study protocol was reviewed and approved by the Institutional Review Board of Landseed International Hospital (IRB No. IRB-19-039-A2) according to the ethical standards stipulated in the 1964 Declaration of Helsinki and its later amendments.

### 2.2. Experimental Design

**Figure 1:**
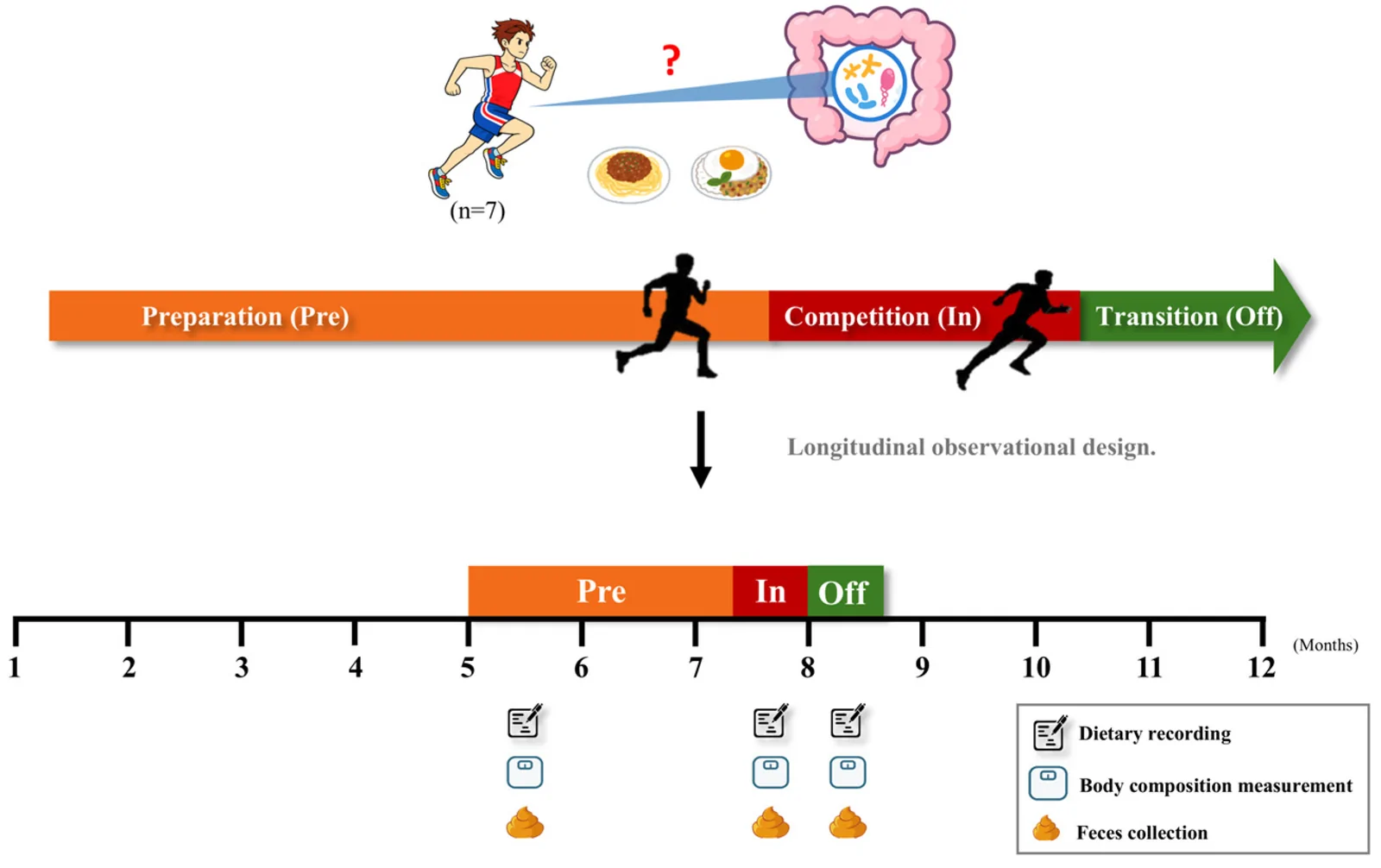
Study design and sample collection across training phases. Seven competitive long-distance runners were enrolled and followed longitudinally across three training phases: preparation (Pre), competition (In), and transition (Off). At each phase, dietary intake and body composition were recorded, and fecal samples were collected.

In this study, we employed a longitudinal observational design (Figure 1), in which the same cohort of competitive long-distance runners was followed across three distinct training phases: (1) preparation (2–3 months before competition), (2) competition (the 3–5 days prior to the competition), and (3) transition (within 3 days after competition). Athletes maintained their habitual training routines throughout the study period, generally completing approximately six training sessions per week, which included sport-specific endurance training and strength training. Detailed external and internal training-load variables, including weekly running volume, running pace, training duration, elevation gain, heart rate, rating of perceived exertion (RPE), and training impulse (TRIMP), were not systematically monitored. Therefore, the predefined preparation, competition, and transition phases were used as the primary framework for longitudinal sampling, but the actual training volume and intensity experienced by each athlete could not be quantitatively characterized. They were instructed to follow their habitual dietary and lifestyle practices, except for avoiding probiotic or lactic acid bacteria supplements. The use of medications and dietary supplements was documented. At each phase, all measurements were conducted, including body composition assessment and fecal sample collection, following an overnight fast of at least 8 h. In addition, participants were required to complete a 3-day dietary record within each phase.

### 2.3. Body Composition

Basic demographic information (age and sex) was collected. Body composition was assessed using a bioelectrical impedance analyzer (InBody 570, Biospace, Inc., Seoul, Republic of Korea) following an overnight fast of at least eight hours.

### 2.4. Dietary Intake Assessment

Participants photographed all foods, beverages, and supplements they consumed; for packaged foods, the brand, weight, and nutrition label information was also recorded. Before the study, participants received standardized instructions and were asked to maintain their habitual dietary and training practices during each recording period. The 3-day dietary records were intended to characterize habitual free-living dietary intake within each training phase rather than intake on specific types of training or non-training days. The recording periods were therefore not standardized to contain an identical number of training and non-training days across participants or phases.

A nutritionist converted dietary records into daily energy and macronutrient intake (carbohydrates, proteins, and fats) using the Taiwan Food Nutrition Database provided by the Ministry of Health and Welfare (MOHW, Taipei, Taiwan) [18], and a registered dietitian verified all entries. Only energy and macronutrient variables were analyzed because these were used to characterize dietary intake patterns potentially relevant to gut microbiota composition.

Dietary intake and gut microbiota analyses focused on the preparation vs. competition and preparation vs. transition comparisons, based on the phase-related differences observed in energy and macronutrient intake. These comparisons were selected to explore whether the dietary patterns accompanying these phase-related changes were also associated with differences in gut microbiota composition.

To characterize dietary intake patterns within these comparisons, k-means clustering was applied to the Z-scored intake values for daily energy, carbohydrates, and proteins. These variables were selected to represent overall dietary energy intake together with two nutritional dimensions of established relevance to endurance training. Carbohydrate intake is closely related to glycogen availability and endurance exercise, whereas protein intake contributes to post-exercise repair, remodeling, and recovery [19,20,21]. Fat intake was assessed and reported as part of the dietary evaluation but was not included as an additional clustering variable to limit redundancy associated with the inclusion of total energy together with all three macronutrients. The elbow method supported a two-cluster solution (k = 2).

For the preparation vs. competition comparison, 14 phase-specific dietary observations derived from the seven athletes were included in the clustering analysis. G1 (*n* = 8 observations) represented a high-intake pattern (2064.7 kcal/day, 4.5 g/kg/day carbohydrate, 1.5 g/kg/day protein), whereas G2 (*n* = 6 observations) represented a low-intake pattern (1074.9 kcal/day, 2.5 g/kg/day carbohydrate, 0.9 g/kg/day protein). Likewise, for the preparation vs. transition comparison, 14 phase-specific dietary observations were included, with G1 (*n* = 8 observations) reflecting a high-intake pattern (1880.9 kcal/day, 3.7 g/kg/day carbohydrate, 1.3 g/kg/day protein) and G2 (*n* = 6 observations) reflecting a low-intake pattern (1052.7 kcal/day, 2.5 g/kg/day carbohydrate, 0.9 g/kg/day protein). Thus, the reported cluster sizes represent athlete-phase observations rather than numbers of unique participants. Associations between dietary intake clusters and gut microbiota diversity and composition were then examined. Because dietary intake differed systematically across training phases, cluster membership overlapped substantially with phase. Therefore, the clustering analyses should be considered exploratory and descriptive and were not intended to estimate dietary effects independent of the training phase.

### 2.5. Fecal Specimen Collection

For each study phase, participants collected one fecal sample following an overnight fast (≥8 h). Samples were collected using sterile tubes prefilled with DNA/RNA Shield (Zymo Research, Irvine, CA, USA). Approximately 0.5 g of stool was transferred into the tube, thoroughly mixed with the preservation solution, and transported to the laboratory. All samples were self-collected at home and delivered on the same day. Upon receipt, samples were immediately stored at −80 °C. The interval between defecation and freezing did not exceed 24 h. Sample collection and handling procedures were performed as previously described in other work [17].

### 2.6. DNA Extraction

Fecal DNA was extracted using a modified bead-beating and phenol–chloroform method originally described by Zhu Heng et al. [22] and further optimized in our previous study [17]. Briefly, fecal samples were suspended in phosphate-buffered saline (PBS) and homogenized. The homogenized samples were subjected to mechanical disruption using glass beads, followed by chemical lysis and phase separation. DNA was precipitated using isopropanol, washed with 70% ethanol, air-dried, and resuspended in ddH_2_O. The purified DNA was stored at −20 °C until further analysis.

### 2.7. 16S rRNA Gene Sequencing and Bioinformatics Processing

The V3-V4 region of the 16S rRNA gene was amplified using specific primers (341F: 5′-CCTACGGGNGGCWGCAG-3′ and 805R: 5′-GACTACHVGGGTATCTAATCC-3′), following the 16S Metagenomic Sequencing Library Preparation protocol (Illumina, Inc., San Diego, CA, USA). Amplicon libraries were sequenced on the Illumina MiSeq™ platform (Illumina, Inc., San Diego, CA, USA).

Bioinformatic analyses were conducted using QIIME2 (version 2021.2; https://qiime2.org) as previously described [23]. Based on the compositional characteristics of microbial community data, correlation networks among bacterial families within each group were constructed using SparCC (version 0.1.0; https://bitbucket.org/yonatanf/sparcc, accessed on 10 August 2026) [24]. The resulting networks were visualized using Cytoscape (version 3.8.2; https://github.com/cytoscape/cytoscape/releases/3.8.2/, accessed on 10 August 2026).

### 2.8. Statistical Analysis

Continuous data are presented as the mean ± standard deviation (SD). Body composition, energy intake, and macronutrient intake were compared across the three training phases using one-way repeated-measures analysis of variance (ANOVA), with athlete treated as the repeated-measures unit. Normality was assessed using the Shapiro–Wilk test alongside a visual inspection of the Q–Q plots. Sphericity was assessed using Mauchly’s test, and Greenhouse–Geisser correction was applied when the assumption of sphericity was violated. When a significant overall effect of the training phase was identified, pairwise comparisons with Bonferroni adjustment were performed. These analyses were conducted using SPSS Statistics for Windows, Version 20.0 (IBM Corp., Armonk, NY, USA).

Taxon-level relative abundances across training phases were evaluated using Friedman tests to account for repeated observations from the same athletes. Benjamini–Hochberg false discovery rate (FDR) correction was applied across taxa at each taxonomic level to control for multiple testing. Taxa with an unadjusted *p* < 0.05 that did not remain significant after FDR correction were regarded as nominal exploratory findings. For taxa showing a significant overall phase effect after FDR correction, post hoc pairwise comparisons were performed using paired Wilcoxon signed-rank tests with Bonferroni adjustment.

Taxon-level differences between dietary intake clusters were evaluated using Wilcoxon rank-sum tests, with Benjamini–Hochberg FDR correction applied across taxa at each taxonomic level. Findings that were significant only before FDR correction were regarded as nominal exploratory findings. Because multiple phase-specific observations could be contributed by the same athlete and cluster membership substantially overlapped with training phase, these cluster-based comparisons are considered exploratory and descriptive and should not be interpreted as evidence of an independent dietary effect.

Alpha-diversity metrics, including Observed Species and the Shannon index, were calculated using QIIME2 (version 2021.2; https://qiime2.org). Differences in alpha diversity across training phases were evaluated using the Kruskal–Wallis test, with Wilcoxon rank-sum tests used for pairwise comparisons, whereas differences between dietary intake clusters were evaluated using Wilcoxon rank-sum tests. Because repeated-measures reanalysis of the original alpha-diversity data was not possible with the available data, these analyses were considered exploratory, and the corresponding *p* values were treated as unadjusted *p* values.

Beta diversity was assessed using weighted and unweighted UniFrac distances and visualized via principal coordinates analysis (PCoA). Group-level differences in microbial community structure across training phases and dietary intake clusters were evaluated using permutational multivariate analysis of variance (PERMANOVA). Because repeated-measures reanalysis of the original beta-diversity distance data was not possible with the available data, these analyses are considered exploratory, and the reported *p* values are treated as unadjusted *p* values.

Differentially abundant genera were identified using DESeq2 (version 1.46.0) in R (version 4.4.3; R Foundation for Statistical Computing, Vienna, Austria). To reduce the influence of sparsely detected taxa, genera present in <25% of samples were excluded before analysis. For longitudinal comparisons across training phases, participant identity was included as a blocking factor in the DESeq2 design matrix (design = ~Athlete_ID + Phase) to account for repeated observations from the same athlete. For differential abundance comparisons between dietary intake clusters, participant identity was similarly included as a blocking factor (design = ~Athlete_ID + Cluster). Wald tests were used for pairwise contrasts, and statistical significance was defined as a Benjamini–Hochberg-adjusted *p* value (*padj*) < 0.05.

Dietary intake patterns were classified using k-means clustering based on the standardized (Z-score) total energy, carbohydrate, and protein intake. For each pairwise phase comparison, 14 phase-specific dietary observations derived from seven athletes were included in the clustering analysis; thus, cluster sample sizes represent athlete–phase observations rather than unique participants. The elbow method was used to determine the number of clusters. Because cluster membership substantially overlapped with training phase, cluster-based microbiome findings should be interpreted as exploratory and descriptive rather than as evidence of an independent dietary effect. A nominal significance level of *p* < 0.05 was used for unadjusted exploratory analyses, whereas FDR-adjusted analyses were considered statistically significant at *padj* < 0.05.

## 3. Results

### 3.1. Characteristics of the Study Population

Table S1 presents the characteristics of the study participants. A total of 7 long-distance runners (5 males, 2 females) were included in this study. Body weight differed significantly across the three training phases (F(2, 12) = 11.196, *p* = 0.002, ηp^2^ = 0.651), exhibiting a significantly higher body weight during the competition phase than during the preparation phase (*p* = 0.021). Absolute skeletal muscle mass also showed a significant overall phase effect (F(2, 12) = 4.166, *p* = 0.042, ηp^2^ = 0.410), although no significant differences were identified in the pairwise comparisons. In contrast, skeletal muscle mass percentage, fat mass, and fat mass percentage did not differ significantly across training phases (*p* > 0.05).

### 3.2. Macronutrient and Energy Intake Across Training Phases

Table 1 summarizes the average dietary intake across three-day periods during each training phase. Greenhouse–Geisser-corrected results are reported where the assumption of sphericity was violated. Significant differences were observed in daily total energy intake per kilogram of body weight across the three phases (F(1.141, 6.849) = 11.435, *p* = 0.011, ηp^2^ = 0.656, ε = 0.571). Bonferroni-adjusted pairwise comparisons showed that energy intake was significantly higher during the competition and transition phases than during the preparation phase (*p* = 0.014 and *p* = 0.001, respectively). Protein (F(2, 12) = 5.910, *p* = 0.016, ηp^2^ = 0.496) and fat intake (F(1.142, 6.853) = 17.224, *p* = 0.004, ηp^2^ = 0.742, ε = 0.571) differed significantly across training phases. Pairwise comparisons showed that protein intake was significantly higher during the competition phase than during the preparation phase (*p* = 0.019), whereas fat intake was significantly higher during both the competition and transition phases than during the preparation phase (*p* < 0.001 and *p* = 0.008, respectively). However, carbohydrate intake did not differ significantly across training phases (F(2, 12) = 3.856, *p* = 0.051, ηp^2^ = 0.391).

The percentage of energy intake from fat differed significantly across training phases (F(2, 12) = 4.150, *p* = 0.043, ηp^2^ = 0.409) and was numerically higher during the competition phase than during the preparation phase. However, the pairwise comparison between these phases was not statistically significant. Conversely, the percentage of energy intake from carbohydrates and protein tended to be lower during the competition phase, but this difference was also not statistically significant (carbohydrate: F(2, 12) = 1.329, *p* = 0.301, ηp^2^ = 0.181; protein: F(2, 12) = 0.331, *p* = 0.724, ηp^2^ = 0.052).

### 3.3. Gut Microbiota Composition Across Training Phases

**Figure 2:**
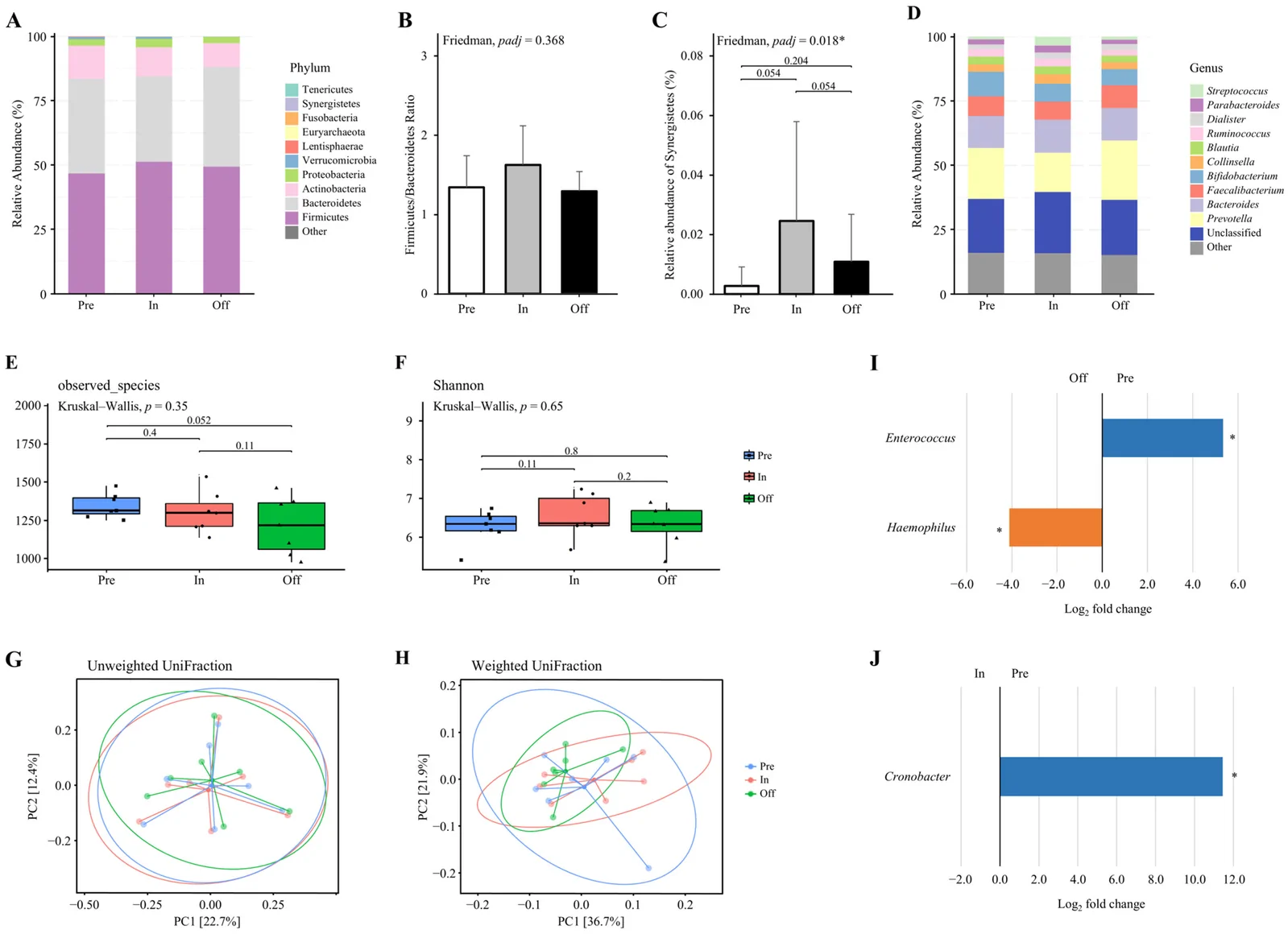
Gut microbiome diversity and composition across training phases. (**A**,**D**) Relative abundance of the 10 most abundant classified microbial taxa at the (**A**) phylum and (**D**) genus levels, with unclassified sequences shown separately and the remaining taxa grouped as Other, across the three training phases: preparation (Pre, *n* = 7), competition (In, *n* = 7), and transition (Off, *n* = 7). (**B**) Firmicutes-to-Bacteroidetes (F/B) ratio across training phases. (**C**) Relative abundance of *Synergistetes* at the phylum level; the overall phase effect was evaluated using the Friedman test with Benjamini–Hochberg false discovery rate (FDR) correction across taxa, and pairwise comparisons were performed using paired Wilcoxon signed-rank tests with Bonferroni adjustment. (**E**,**F**) Alpha diversity assessed according to the observed species and Shannon index using Kruskal–Wallis-based analysis. (**G**,**H**) Beta diversity visualized via principal coordinate analysis (PCoA) based on unweighted and weighted UniFrac distances; percentages on the axes indicate the explained variance of PC1 and PC2. Alpha- and beta-diversity results are presented for exploratory purposes only. (**I**,**J**) Differentially abundant genera identified using DESeq2 between (**I**) the preparation and transition phases (Pre vs. Off) and (**J**) the preparation and competition phases (Pre vs. In). For panels (**B**,**C**,**I**,**J**), *padj* denotes Benjamini–Hochberg-adjusted *p* values. Values shown for pairwise comparisons in panel (**C**) are Bonferroni-adjusted *p* values. For panels (**E**,**H**), *p* denotes unadjusted *p* values. For FDR-adjusted results, * *padj* < 0.05; for unadjusted analyses, * *p* < 0.05.

Figure 2A,D illustrate the relative abundance of gut microbiota across the three training phases at the phylum and genus levels, respectively.

At the phylum level, the dominant phyla included *Firmicutes*, *Bacteroidetes*, *Actinobacteria*, *Proteobacteria*, and *Verrucomicrobia* (Figure 2A). The Firmicutes-to-Bacteroidetes (F/B) ratio did not differ significantly across the training phases (Friedman test, *padj* = 0.368; Figure 2B). Among the phyla examined, *Synergistetes* showed a significant overall phase effect after Benjamini–Hochberg false discovery rate correction (Friedman test, *padj* = 0.018; Figure 2C). However, subsequent paired Wilcoxon signed-rank tests with Bonferroni adjustment showed no significant pairwise differences among the preparation, competition, and transition phases (all *padj* > 0.05).

At the genus level, the dominant genera were *Prevotella*, *Bacteroides*, *Faecalibacterium*, *Bifidobacterium*, *Collinsella*, and *Blautia* (Figure 2D). Exploratory Friedman analyses identified nominal phase-related differences in *Haemophilus*, *Roseburia*, *Dehalobacterium*, *Christensenella*, *Finegoldia*, *Clostridium*, *Burkholderia*, and *Streptococcus* (unadjusted *p* < 0.05); however, none remained statistically significant after Benjamini–Hochberg FDR correction (all *padj* > 0.05).

### 3.4. Alpha- and Beta-Diversity Analysis Across Training Phases

Alpha- and beta-diversity analyses are presented descriptively across the three training phases (Figure 2E–H). The original analyses yielded unadjusted *p* values > 0.05 for observed species, the Shannon index, weighted UniFrac, and unweighted UniFrac. Because these tests did not account for repeated observations within athletes, these values are reported only as exploratory outputs and should not be interpreted as evidence for either the presence or absence of differences across phases.

### 3.5. Taxonomic Abundance Changes Across Training Phases

Differential abundance analysis identified several genus-level differences after Benjamini–Hochberg correction (Figure 2I,J). In the transition-versus-preparation comparison, *Haemophilus* was significantly more abundant during the transition phase, whereas *Enterococcus* was significantly more abundant during the preparation phase (*padj* < 0.05; Figure 2I). In the competition-versus-preparation comparison, *Cronobacter* was significantly more abundant during the preparation phase (*padj* < 0.05; Figure 2J). No genera showed significant differences between the competition and transition phases after multiple-testing correction. Although several additional genera displayed large absolute log_2_ fold changes (>2.0), these did not reach statistical significance after adjustment and are reported in Tables S2–S4.

### 3.6. Exploratory Gut Microbiota Comparisons Across Dietary Intake Clusters in the Preparation–Competition Comparison

**Figure 3:**
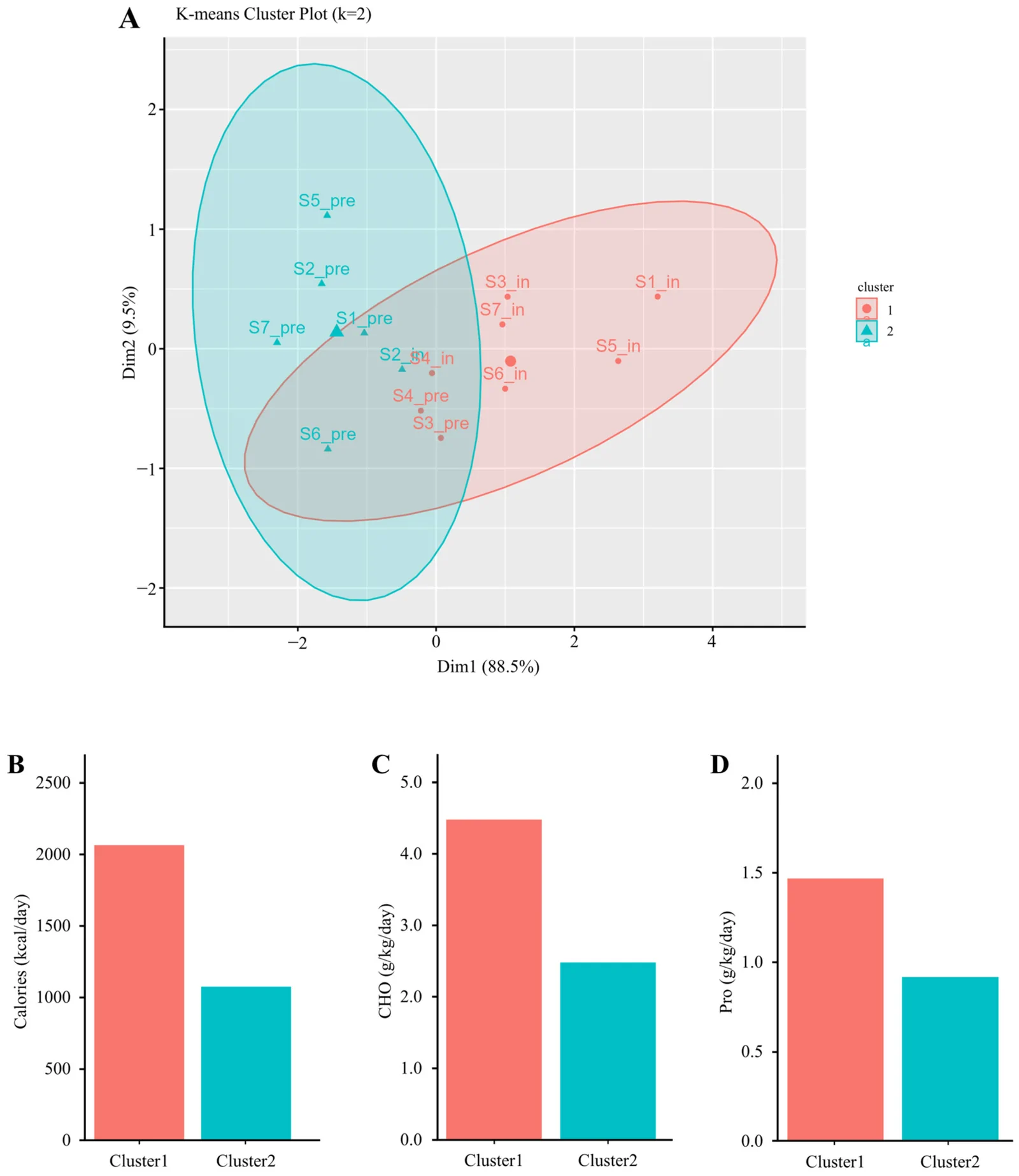
K-means clustering of phase-specific dietary intake observations during the preparation and competition phases. (**A**) K-means cluster plot (k = 2) visualizing two distinct dietary intake patterns derived from standardized (Z-scored) daily energy, carbohydrate, and protein intake. A total of 14 phase-specific observations were included, comprising one preparation-phase and one competition-phase observation from each of the seven athletes. Each point therefore represents an athlete–phase observation rather than a unique participant. (**B**–**D**) Dietary intake characteristics of the two clusters, including (**B**) total energy intake (kcal/day), (**C**) carbohydrate intake (g/kg/day), and (**D**) protein intake (g/kg/day). Cluster 1 (G1; *n* = 8 observations) was characterized by higher energy, carbohydrate, and protein intake, whereas Cluster 2 (G2; *n* = 6 observations) was characterized by the lower intake of these variables. During the preparation phase, 2 observations were assigned to G1 and 5 to G2, whereas during the competition phase, 6 observations were assigned to G1 and 1 to G2. Cluster sample sizes refer to phase-specific observations, not unique athletes.

Gut microbiota profiles were examined in relation to dietary intake clusters derived from the preparation and competition phases. A total of 14 phase-specific observations from seven athletes were included in the clustering analysis. G1 comprised eight observations and represented a higher-intake pattern (2064.7 kcal/day, 4.5 g/kg/day carbohydrate, and 1.5 g/kg/day protein), whereas G2 comprised six observations and represented a lower-intake pattern (1074.9 kcal/day, 2.5 g/kg/day carbohydrate, and 0.9 g/kg/day protein) (Figure 3A–D). During the preparation phase, two observations were assigned to G1 and five to G2, whereas during the competition phase, six observations were assigned to G1 and one to G2. This distribution demonstrates substantial correspondence between dietary cluster membership and the training phase. Therefore, the following microbiome comparisons were considered exploratory and descriptive and were not intended to isolate an independent dietary effect.

**Figure 4:**
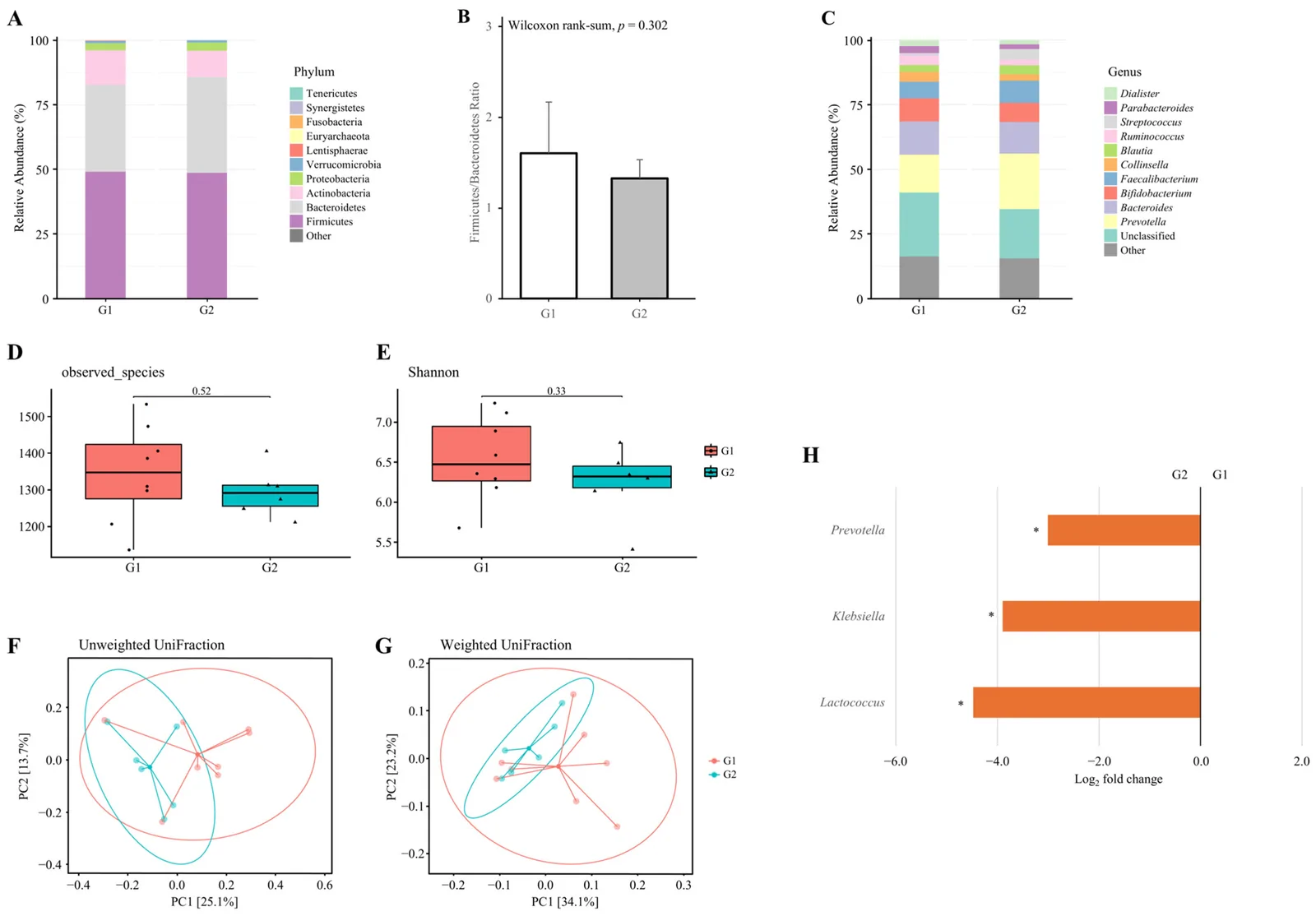
Gut microbiome diversity and composition across dietary intake clusters in the preparation–competition comparison. (**A**,**C**) Relative abundance of the 10 most abundant classified microbial taxa at the (**A**) phylum and (**C**) genus levels across the two dietary intake clusters, with unclassified sequences shown separately and the remaining taxa grouped as Other. Cluster 1 (G1; *n* = 8 observations) represents the higher-intake pattern, whereas Cluster 2 (G2; *n* = 6 observations) represents the lower-intake pattern. Cluster sample sizes refer to phase-specific observations rather than unique athletes. (**B**) Firmicutes-to-Bacteroidetes (F/B) ratio between dietary intake clusters, evaluated using the Wilcoxon rank-sum test. (**D**,**E**) Alpha diversity assessed according to the observed species and Shannon index. (**F**,**G**) Beta diversity visualized via principal coordinate analysis (PCoA) based on unweighted and weighted UniFrac distances; percentages on the axes indicate the explained variance of PC1 and PC2. Alpha- and beta-diversity results are presented for exploratory purposes only. (**H**) Differentially abundant genera identified using DESeq2 between G1 and G2. For panels (**B**,**D**–**G**), *p* denotes unadjusted *p* values. For panel (**H**), *padj* denotes Benjamini–Hochberg-adjusted *p* values. For the FDR-adjusted results, * *padj* < 0.05; for unadjusted analyses, * *p* < 0.05.

Figure 4A,C show the relative abundance of gut microbiota across clusters. The dominant phyla and genera were consistent with those identified across training phases (Section 3.3), with *Firmicutes*, *Bacteroidetes*, *Actinobacteria*, *Proteobacteria*, and *Verrucomicrobia*, and genera such as *Prevotella*, *Bacteroides*, *Faecalibacterium*, *Bifidobacterium*, *Collinsella*, and *Blautia* prevailing across clusters. The Firmicutes-to-Bacteroidetes (F/B) ratio did not differ significantly across dietary intake clusters (Wilcoxon rank-sum, *p* = 0.302; Figure 4B). Although several phylum- and genus-level taxa showed nominal differences between clusters in the exploratory Wilcoxon analyses, none remained statistically significant after Benjamini–Hochberg FDR correction.

Alpha- and beta-diversity analyses are presented descriptively for the preparation–competition dietary clusters (Figure 4D–G). The original analyses yielded unadjusted *p* values > 0.05 for observed species, the Shannon index, weighted UniFrac, and unweighted UniFrac. Because these tests did not account for repeated observations within athletes, these values are reported only as exploratory outputs and should not be interpreted as evidence for either the presence or absence of differences between clusters.

In the exploratory DESeq2 comparison, *Prevotella*, *Klebsiella*, and *Lactococcus* differed significantly between G1 and G2 after Benjamini–Hochberg correction (all *padj* < 0.05; Figure 4H). These genera showed lower abundance in G1 relative to G2, as indicated by negative log2 fold changes. Although several additional genera displayed large absolute log2 fold changes (>2.0), these did not reach statistical significance after adjustment and are reported in Table S5. Given the substantial overlap between cluster membership and training phase, these differences reflect cluster-associated patterns within the combined dietary–training context and should not be interpreted as independent dietary effects.

### 3.7. Exploratory Gut Microbiota Comparisons Across Dietary Intake Clusters in the Preparation–Transition Comparison

**Figure 5:**
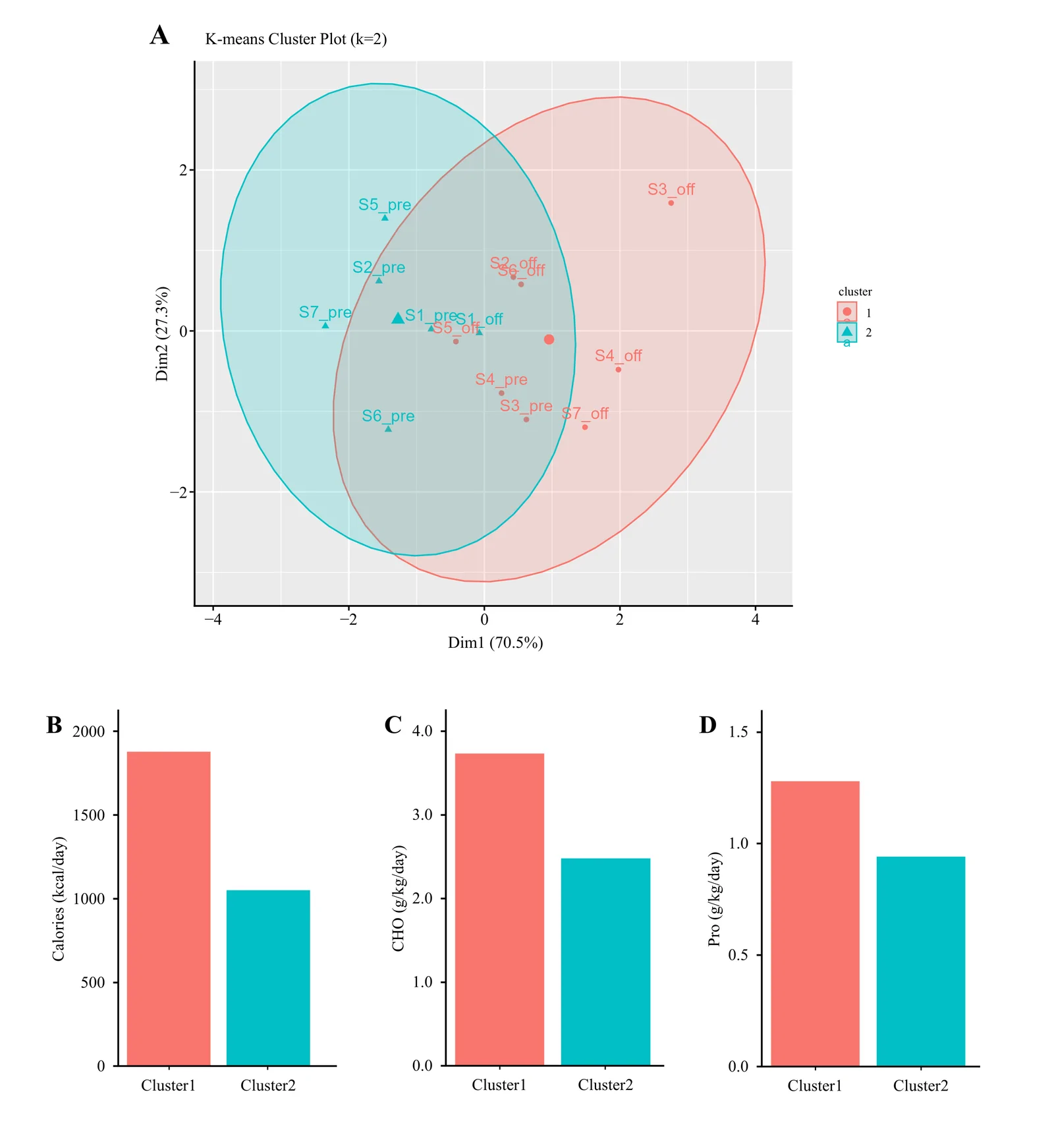
K-means clustering of phase-specific dietary intake observations during the preparation and transition phases. (**A**) K-means cluster plot (k = 2) visualizing two distinct dietary intake patterns derived from standardized (Z-scored) daily energy, carbohydrate, and protein intake. A total of 14 phase-specific observations were included, comprising one preparation-phase and one transition-phase observation from each of the seven athletes. Each point therefore represents an athlete–phase observation rather than a unique participant. (**B**–**D**) Dietary intake characteristics of the two clusters, including (**B**) total energy intake (kcal/day), (**C**) carbohydrate intake (g/kg/day), and (**D**) protein intake (g/kg/day). Cluster 1 (G1; *n* = 8 observations) was characterized by a higher energy, carbohydrate, and protein intake, whereas Cluster 2 (G2; *n* = 6 observations) was characterized by a lower intake of these variables. During the preparation phase, 2 observations were assigned to G1 and 5 to G2, whereas during the transition phase, 6 observations were assigned to G1 and 1 to G2. Cluster sample sizes refer to phase-specific observations, not unique athletes.

Gut microbiota profiles were also examined in relation to dietary intake clusters derived from the preparation and transition phases. A total of 14 phase-specific observations from seven athletes were included. G1 comprised eight observations and represented a higher-intake pattern (1880.9 kcal/day, 3.7 g/kg/day carbohydrate, and 1.3 g/kg/day protein), whereas G2 comprised six observations and represented a lower-intake pattern (1052.7 kcal/day, 2.5 g/kg/day carbohydrate, and 0.9 g/kg/day protein) (Figure 5A–D). During the preparation phase, two observations were assigned to G1 and five to G2, whereas during the transition phase, six observations were assigned to G1 and one to G2. This distribution similarly demonstrates substantial correspondence between dietary cluster membership and training phase. Accordingly, the following microbiome comparisons should be considered exploratory and descriptive rather than evidence of an independent dietary effect.

**Figure 6:**
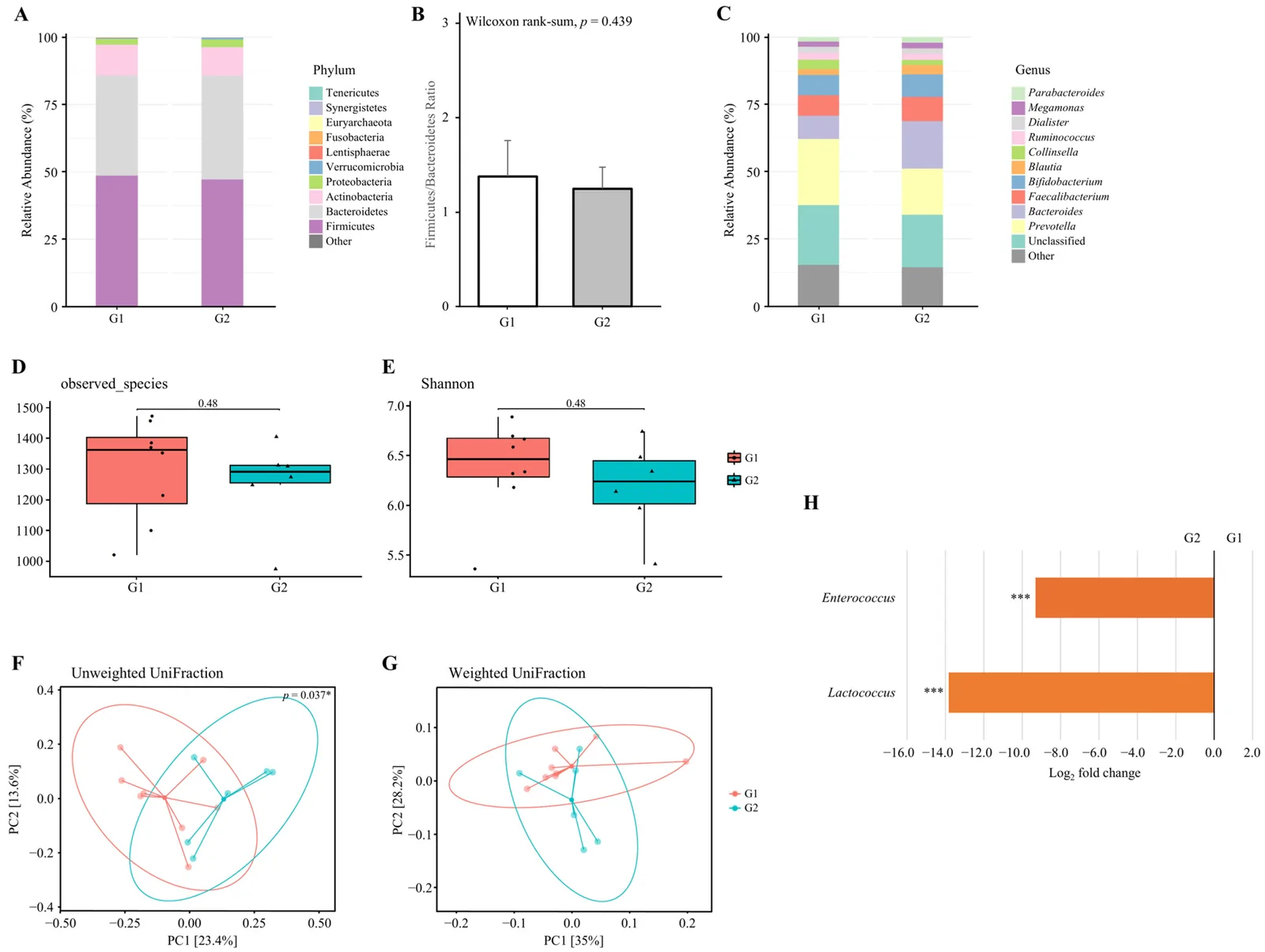
Gut microbiome diversity and composition across dietary intake clusters in the preparation–transition comparison. (**A**,**C**) Relative abundance of the 10 most abundant classified microbial taxa at the (**A**) phylum and (**C**) genus levels across the two dietary intake clusters, with unclassified sequences shown separately and the remaining taxa grouped as Other. Cluster 1 (G1; *n* = 8 observations) represented the higher-intake pattern, whereas Cluster 2 (G2; *n* = 6 observations) represented the lower-intake pattern. Cluster sample sizes refer to phase-specific observations rather than unique athletes. (**B**) Firmicutes-to-Bacteroidetes (F/B) ratio between dietary intake clusters, evaluated using the Wilcoxon rank-sum test. (**D**,**E**) Alpha diversity assessed according to the observed species and Shannon index. (**F**,**G**) Beta diversity visualized using principal coordinate analysis (PCoA) based on unweighted and weighted UniFrac distances; percentages on the axes indicate the explained variance of PC1 and PC2. Alpha- and beta-diversity results are presented for exploratory purposes only. (**H**) Differentially abundant genera identified using DESeq2 between G1 and G2. For panels (**B**,**D**–**G**), *p* denotes unadjusted *p* values. For panel (**H**), *padj* denotes Benjamini–Hochberg-adjusted *p* values. For FDR-adjusted results, * *padj* < 0.05 and *** *padj* < 0.001; for unadjusted analyses, * *p* < 0.05 and *** *p* < 0.001.

Figure 6A,C show the relative abundance of gut microbiota across clusters. The dominant phyla and genera were consistent with those identified across training phases (Section 3.3), with *Firmicutes*, *Bacteroidetes*, *Actinobacteria*, *Proteobacteria*, and *Verrucomicrobia* and genera such as *Prevotella*, *Bacteroides*, *Faecalibacterium*, *Bifidobacterium*, *Collinsella*, and *Blautia* prevailing across clusters. The Firmicutes-to-Bacteroidetes (F/B) ratio did not differ significantly across dietary intake clusters (Wilcoxon rank-sum, *p* = 0.439; Figure 6B). Although several taxa showed nominal differences between clusters in the exploratory Wilcoxon analyses, none remained statistically significant after Benjamini–Hochberg FDR correction.

Alpha- and beta-diversity analyses are presented descriptively for the preparation–transition dietary clusters (Figure 6D–G). The original analyses yielded unadjusted *p* values > 0.05 for observed species, the Shannon index, and weighted UniFrac, and an unadjusted *p* value of 0.037 for unweighted UniFrac. Because these tests did not account for repeated observations within athletes, these values are reported only as exploratory outputs and should not be interpreted as evidence for either the presence or absence of differences between clusters.

In the exploratory DESeq2 comparison, *Enterococcus* and *Lactococcus* differed significantly between G1 and G2 after Benjamini–Hochberg correction (both *padj* < 0.05; Figure 6H). Both genera showed lower abundance in G1 relative to G2, as indicated by negative log_2_ fold changes. Although several additional genera displayed large absolute log_2_ fold changes (>2.0), these did not reach statistical significance after adjustment and are reported in Table S6. Because cluster membership substantially overlapped with training phase, these findings were interpreted as cluster-associated patterns within the combined dietary–training context rather than as independent dietary effects.

## 4. Discussion

This longitudinal study characterized changes in dietary intake and gut microbiota composition across three training phases in competitive long-distance runners and explored associations between dietary intake patterns and microbial characteristics. Body composition was generally stable across the training cycle, although modest phase-related changes in body weight and absolute skeletal muscle mass were observed. Energy, protein, and fat intake differed across training phases, with generally higher values observed during the competition phase. Carbohydrate intake also showed a higher mean value during the competition phase, although the overall phase effect did not reach statistical significance. This pattern is broadly consistent with nutritional periodization strategies that emphasize greater carbohydrate availability to support glycogen restoration and endurance performance [11,13].

Exploratory alpha- and beta-diversity analyses yielded no statistically detectable differences across training phases. Because these analyses did not account for repeated observations within athletes, the associated *p* values are reported for exploratory purposes only and are not used to infer the presence or absence of community-level microbiome changes. Accordingly, no conclusions regarding microbiome stability or resilience are drawn from these analyses. As a contextual comparison, Craven et al. reported that changes in training volume in highly trained middle-distance runners did not significantly alter alpha diversity or global microbial composition, while several lower-level taxa varied across high-volume training and tapering periods [25]. These previous findings provide relevant context for examining taxon-specific responses to training-related changes but should not be considered as confirmation of this study’s community-level findings.

After correction for multiple testing, the evidence for widespread taxonomic changes across phases was limited. Although several genera showed nominal differences in the Friedman analyses, none remained significant after FDR correction at the genus level. In contrast, the participant-blocked DESeq2 analysis identified a small number of genera that remained significant after Benjamini–Hochberg adjustment. Among these, *Haemophilus* is particularly noteworthy because its abundance differed between the preparation and transition phases and because previous work on endurance runners has also suggested that this genus may respond to changes in training load. Craven et al. reported reductions in both *Haemophilus* and *H. parainfluenzae* following a high-volume training block in highly trained middle-distance runners, despite no significant change in dietary intake [25]. Although the direction and timing of these changes are not directly comparable with the higher *Haemophilus* abundance observed during the transition phase in this study, together, these findings support *Haemophilus* as a candidate phase- or training-responsive taxon rather than establish it plays a specific beneficial or detrimental role in endurance performance. In addition, Jang et al. reported sport- and diet-related differences in *Haemophilus* abundance among bodybuilders, distance runners, and sedentary controls [26], further indicating that its abundance may reflect the combined influence of training modality and nutritional exposure rather than a single isolated factor.

Nevertheless, the biological interpretation of the findings regarding *Haemophilus* should remain cautious. Although *Haemophilus* remained significant after multiple-testing correction in the preparation–transition comparison, the present observational design does not establish whether this change reflects a direct effect of the training phase, dietary intake, recovery status, or other phase-related physiological factors. Previous studies have also reported changes in *Haemophilus* abundance in endurance-trained athletes following alterations in training volume, suggesting that this genus may be responsive to changes in the training environment. However, the functional implications of this variation in endurance athletes remain unclear. Therefore, the present finding should be regarded as a preliminary phase-associated microbial signal that warrants confirmation in larger longitudinal cohorts.

Two additional DESeq2 findings, *Enterococcus* and *Cronobacter*, also require cautious interpretation. *Enterococcus* was more abundant during the preparation phase than the transition phase. In a previous study by our group involving competitive weightlifters, *Enterococcus* abundance was found to be similarly greater during the preparation phase than the competition phase [17]. Although the physiological and nutritional demands of weightlifting and endurance running differ considerably, the observation of a similar preparation-phase pattern across two distinct athlete cohorts suggests that *Enterococcus* may be sensitive to changes in the broader training-phase environment. Nevertheless, the biological interpretation of this genus remains complex because *Enterococcus* includes both commensal and opportunistic strains, particularly within *E. faecalis* and *E. faecium* [27]. Thus, the observed increase should not be interpreted as inherently beneficial or detrimental. Rather, it may reflect combined changes in training, dietary intake, recovery status, and other phase-related factors that could not be disentangled in this study.

*Cronobacter* was more abundant during the preparation than the competition phase. However, evidence linking this genus to endurance training or athletic performance remains sparse. Although *Cronobacter* species can be detected in the adult gastrointestinal tract, several species are primarily studied in the context of environmental persistence and opportunistic infection [28], and the detection of a genus-level signal in healthy athletes should not be interpreted as evidence of pathogenicity. Accordingly, this finding should be considered preliminary and warrants confirmation at the species and strain levels in larger longitudinal cohorts.

The dietary clustering analyses should be interpreted even more cautiously because cluster membership closely corresponded with training phase. Nevertheless, the DESeq2 findings within these exploratory comparisons may provide hypotheses for future studies. *Lactococcus* was identified in both cluster comparisons, which is noteworthy because Bielik et al. observed an increase in *Lactococcus* following seven weeks of high-intensity training in competitive swimmers, even in athletes who did not receive the probiotic intervention [29]. This finding suggests that *Lactococcus* may respond to the training environment itself, illustrating why the present cluster-associated difference cannot be attributed solely to nutritional intake.

*Prevotella*, identified in the preparation–competition cluster comparison, has been repeatedly reported in endurance-oriented athlete microbiomes. Studies on competitive cyclists have associated greater *Prevotella* abundance with microbial pathways involved in carbohydrate and amino acid metabolism, and systematic reviews of athlete microbiome studies have highlighted *P. copri* as a taxon of potential metabolic relevance in exercise settings [30,31]. However, the present finding should not be interpreted as evidence that higher or lower carbohydrate intake directly altered *Prevotella*, because the dietary clusters largely recapitulated the training phase. Indeed, the inability to separate dietary exposure from the surrounding training context is a central limitation of these analyses. The same consideration applies to *Klebsiella*, *Enterococcus*, and *Lactococcus*: their between-cluster differences are best interpreted as exploratory signatures of the combined nutritional and training environment rather than independent responses to dietary intake.

Taken together, the participant-blocked DESeq2 analysis identified a limited number of genera with phase-related differences after multiple-testing correction. Of these, *Haemophilus* is particularly relevant because previous longitudinal evidence in trained runners also suggests sensitivity to changes in training volume. However, the absence of functional measurements and concurrent changes in training and dietary intake preclude mechanistic interpretation. These taxonomic signals should therefore be considered preliminary candidates for confirmation in larger longitudinal studies integrating objective training-load measures, dietary assessment, and metagenomic or metabolomic analyses.

Several limitations should be considered when interpreting these findings. First, no a priori sample-size calculation was performed, and the small cohort of seven athletes substantially limits statistical power and generalizability, particularly given the high inter- and intra-individual variability of human gut microbiome data. The taxon-level findings and exploratory community-level results require cautious interpretation and confirmation in larger cohorts. Repeated-measures reanalysis of the original alpha- and beta-diversity data was not possible with the available data. The corresponding unadjusted *p* values were therefore retained for exploratory reporting only and were not used to infer community-level changes or microbiome stability. Second, the 3-day dietary recording periods were not standardized according to the number of training and non-training days included. Consequently, day-to-day variation associated with training status may have introduced additional variability into the dietary estimates. The relatively low dietary intake reported during the preparation phase should also be interpreted cautiously because under-reporting cannot be excluded. More importantly, dietary intake and training phase were strongly aligned in this cohort. In both pairwise clustering analyses, 11 of 14 observations followed the predominant pattern of lower intake during preparation and higher intake during competition or transition, leaving insufficient within-phase dietary variation to distinguish the independent contributions of dietary intake and training phase. Accordingly, the cluster-based analyses should be interpreted as exploratory descriptions of microbial patterns accompanying the combined dietary and training context rather than as evidence of independent dietary effects. Although participant identity was included as a blocking factor in the cluster-based DESeq2 models, the exploratory Wilcoxon and diversity analyses did not explicitly model the repeated observations contributed by the same athletes, providing an additional reason for cautious interpretation of these analysis results.

Third, objective external and internal training-load measures were not systematically monitored. Therefore, although participants were followed across predefined preparation, competition, and transition phases, the actual training stimulus experienced by each athlete could not be quantified. As a result, the potential contributions of training volume, intensity, competition-related stress, and recovery to the observed microbial differences could not be separated from other phase-related exposures. Future studies should incorporate standardized monitoring of both external and internal training load to clarify the relationship between training stress and gut microbiome variation. Fourth, the taxonomic analyses were based primarily on relative abundance data obtained from 16S rRNA gene sequencing. Because microbiome relative-abundance data are compositional, an apparent increase or decrease in a given taxon does not necessarily indicate a corresponding change in its absolute microbial abundance. Moreover, 16S rRNA sequencing primarily provides taxonomic information and does not directly characterize microbial functional activity. Functional microbial outcomes, including fecal short-chain fatty acids such as acetate, propionate, and butyrate, were not quantified, and shotgun metagenomic sequencing was not performed. Therefore, we cannot determine whether the observed taxonomic differences were accompanied by corresponding changes in microbial metabolic pathways or metabolite production. Future studies should incorporate larger cohorts, objective external and internal training-load monitoring, greater within-phase dietary variation, absolute microbial quantification, and integrated shotgun metagenomic and metabolomic analyses. Analytical approaches that simultaneously model dietary intake and training phase while accounting for repeated observations within athletes may further help disentangle the respective contributions of dietary intake and training phase to gut microbial variation.

Overall, this study provides a descriptive longitudinal characterization of dietary intake and gut microbiota across different phases of a competitive training cycle in long-distance runners. Energy, protein, and fat intake differed across training phases, and a limited number of taxa showed phase-related differences after multiple-testing correction. Alpha- and beta-diversity results were presented for exploratory purposes only, given the small sample size and the lack of adjustment for repeated observations within athletes. Because dietary intake and training phase changed concurrently, the observed taxonomic differences are best interpreted within the combined nutritional and training environment rather than as independent responses to either factor.

## 5. Conclusions

In conclusion, the energy, protein, and fat intake differed across training phases in competitive long-distance runners, whereas carbohydrate intake did not reach statistical significance. Alpha- and beta-diversity results were presented for exploratory purposes only, given the small sample size and the lack of adjustment for repeated observations within athletes. These analyses establish neither the presence nor absence of community-level differences. After multiple-testing correction, only a limited number of taxonomic differences were identified, including *Haemophilus* and *Enterococcus* between the preparation and transition phases and *Cronobacter* between the preparation and competition phases in the DESeq2 analysis. The dietary cluster analyses were exploratory and strongly aligned with the training phase; therefore, cluster-associated microbial differences cannot be attributed independently to dietary intake. Overall, these findings should be considered preliminary and warrant confirmation in larger longitudinal studies incorporating objective training-load assessment, greater within-phase dietary variation, and functional microbiome measurements.

## Figures and Tables

**Table 1:** Macronutrient and energy intake of long-distance runners during preparation, competition, and transition phases.

	Preparation (Pre) *n* = 7	Competition (In) *n* = 7	Transition (Off) *n* = 7	ηp^2^	*p* Value	Pairwise Comparisons
Carbohydrate (g/day)	158.6 ± 68.6	253.9 ± 59.8	207.1 ± 64.5	0.391	0.051	
Carbohydrate (g/kg/day)	2.8 ± 1.1	4.4 ± 1.1	3.6 ± 0.8	0.392	0.051	
Carbohydrate (% kcal/day)	52.3 ± 11.5	48.1 ± 4.6	44.5 ± 9.5	0.181	0.301	
Fat (g/day)	38.2 ± 10.7	80.8 ± 11.4	81.3 ± 28.2	0.742	0.004 **	pre < in, off
Fat (g/kg/day)	0.7 ± 0.2	1.4 ± 0.3	1.4 ± 0.5	0.729	<0.001 ***	pre < in, off
Fat (% kcal/day)	29.7 ± 6.7	35.3 ± 5.5	39.1 ± 8.0	0.409	0.043 *	NS
Protein (g/day)	51.4 ± 14.8	88.5 ± 23.9	77.1 ± 23.0	0.496	0.016 *	pre < in
Protein (g/kg/day)	0.9 ± 0.2	1.5 ± 0.4	1.3 ± 0.4	0.472	0.022 *	pre < in
Protein (% kcal/day)	18.1 ± 6.1	16.6 ± 2.1	16.4 ± 1.9	0.052	0.724	
Calories (kcal/day)	1183.8 ± 345.8	2097.2 ± 373.8	1868.2 ± 439.1	0.674	0.001 **	pre < in, off
Calories (kcal/kg/day)	21.1 ± 5.5	36.8 ± 8.0	32.6 ± 5.3	0.656	0.011 *	pre < in, off

Data are expressed as the mean ± SD. * *p* < 0.05, ** *p* < 0.01, *** *p* < 0.001. ηp^2^, partial eta squared. Pairwise comparisons were adjusted using the Bonferroni method. Greenhouse–Geisser correction was applied when the assumption of sphericity was violated. NS, no significant pairwise difference.

## Data Availability

Summary results are provided in the article and Supplementary Materials, which do not contain the complete participant-level dataset. The original alpha-diversity data and beta-diversity distance matrices required for repeated-measures reanalysis are no longer available and cannot be provided, preventing independent reanalysis of these data and limiting the reproducibility of these analyses.

## Supplementary Materials

The following supporting information can be downloaded at https://www.mdpi.com/article/10.3390/nu18183036/s1: Table S1: Characteristic of study participants; Table S2: DESeq2 analysis identifying differentially abundant genera between the samples obtained through Pre and In; Table S3: DESeq2 analysis identifying differentially abundant genera between the samples obtained through In and Off; Table S4: DESeq2 analysis identifying differentially abundant genera between the samples obtained through Pre and Off; Table S5: DESeq2 analysis identifying differentially abundant genera between G1 and G2 based on samples obtained during the pre- and in-competition phases; Table S6: DESeq2 analysis identifying differentially abundant genera between G1 and G2 based on samples obtained during the pre- and off-competition phases.

## References

[1] Hooper L.V., Gordon J.I. (2001). Commensal host-bacterial relationships in the gut. Science.

[2] Lee J.-Y., Tsolis R.M., Bäumler A.J. (2022). The microbiome and gut homeostasis. Science.

[3] Liu X., Xu M., Wang H., Zhu L. (2025). Role and Mechanism of Short-Chain Fatty Acids in Skeletal Muscle Homeostasis and Exercise Performance. Nutrients.

[4] Sales K.M., Reimer R.A. (2023). Unlocking a novel determinant of athletic performance: The role of the gut microbiota, short-chain fatty acids, and “biotics” in exercise. J. Sport Health Sci..

[5] Clauss M., Gérard P., Mosca A., Leclerc M. (2021). Interplay Between Exercise and Gut Microbiome in the Context of Human Health and Performance. Front. Nutr..

[6] Mohr A.E., Mach N., Pugh J., Grosicki G.J., Allen J.M., Karl J.P., Whisner C.M. (2025). Mechanisms underlying alterations of the gut microbiota by exercise and their role in shaping ecological resilience. FEMS Microbiol. Rev..

[7] Mach N., Fuster-Botella D. (2017). Endurance exercise and gut microbiota: A review. J. Sport Health Sci..

[8] Scheiman J., Luber J.M., Chavkin T.A., MacDonald T., Tung A., Pham L.D., Wibowo M.C., Wurth R.C., Punthambaker S., Tierney B.T. (2019). Meta-omics analysis of elite athletes identifies a performance-enhancing microbe that functions via lactate metabolism. Nat. Med..

[9] Flint H.J., Duncan S.H., Scott K.P., Louis P. (2015). Links between diet, gut microbiota composition and gut metabolism. Proc. Nutr. Soc..

[10] Schneider E., O’Riordan K.J., Clarke G., Cryan J.F. (2024). Feeding gut microbes to nourish the brain: Unravelling the diet-microbiota-gut-brain axis. Nat. Metab..

[11] Stellingwerff T., Morton J.P., Burke L.M. (2019). A Framework for Periodized Nutrition for Athletics. Int. J. Sport Nutr. Exerc. Metab..

[12] Jeukendrup A.E. (2017). Periodized Nutrition for Athletes. Sports Med..

[13] Burke L.M., Jeukendrup A.E., Jones A.M., Mooses M. (2019). Contemporary Nutrition Strategies to Optimize Performance in Distance Runners and Race Walkers. Int. J. Sport Nutr. Exerc. Metab..

[14] Cao W., He Y., Fu R., Chen Y., Yu J., He Z. (2025). A Review of Carbohydrate Supplementation Approaches and Strategies for Optimizing Performance in Elite Long-Distance Endurance. Nutrients.

[15] Patel B.K., Patel K.H., Lee C.N., Moochhala S. (2024). Intestinal Microbiota Interventions to Enhance Athletic Performance-A Review. Int. J. Mol. Sci..

[16] Furber M.J.W., Young G.R., Holt G.S., Pyle S., Davison G., Roberts M.G., Roberts J.D., Howatson G., Smith D.L. (2022). Gut Microbial Stability is Associated with Greater Endurance Performance in Athletes Undertaking Dietary Periodization. mSystems.

[17] Kuo C.Y., Lo Y.C., Chen W.L., Hsu Y.J. (2025). Phase-Specific Alterations in Gut Microbiota and Their Associations with Energy Intake and Nutritional Clustering in Competitive Weightlifters. Nutrients.

[18] Taiwan Food and Drug Administration Taiwanese Food Composition and Nutrient Database. https://consumer.fda.gov.tw/Food/TFND.aspx?nodeID=178.

[19] Burke L.M., Hawley J.A., Wong S.H., Jeukendrup A.E. (2011). Carbohydrates for training and competition. J. Sports Sci..

[20] Thomas D.T., Erdman K.A., Burke L.M. (2016). Position of the Academy of Nutrition and Dietetics, Dietitians of Canada, and the American College of Sports Medicine: Nutrition and Athletic Performance. J. Acad. Nutr. Diet..

[21] Jäger R., Kerksick C.M., Campbell B.I., Cribb P.J., Wells S.D., Skwiat T.M., Purpura M., Ziegenfuss T.N., Ferrando A.A., Arent S.M. (2017). International Society of Sports Nutrition Position Stand: Protein and exercise. J. Int. Soc. Sports Nutr..

[22] Zhu H., Qu F., Zhu L.H. (1993). Isolation of genomic DNAs from plants, fungi and bacteria using benzyl chloride. Nucleic Acids Res..

[23] Bolyen E., Rideout J.R., Dillon M.R., Bokulich N.A., Abnet C.C., Al-Ghalith G.A., Alexander H., Alm E.J., Arumugam M., Asnicar F. (2019). Reproducible, interactive, scalable and extensible microbiome data science using QIIME 2. Nat. Biotechnol..

[24] Friedman J., Alm E.J. (2012). Inferring correlation networks from genomic survey data. PLoS Comput. Biol..

[25] Craven J., Cox A.J., Bellinger P., Desbrow B., Irwin C., Buchan J., McCartney D., Sabapathy S. (2022). The influence of exercise training volume alterations on the gut microbiome in highly-trained middle-distance runners. Eur. J. Sport Sci..

[26] Jang L.G., Choi G., Kim S.W., Kim B.Y., Lee S., Park H. (2019). The combination of sport and sport-specific diet is associated with characteristics of gut microbiota: An observational study. J. Int. Soc. Sports Nutr..

[27] Krawczyk B., Wityk P., Gałęcka M., Michalik M. (2021). The Many Faces of Enterococcus spp.-Commensal, Probiotic and Opportunistic Pathogen. Microorganisms.

[28] Feeney A., Kropp K.A., O’Connor R., Sleator R.D. (2014). Cronobacter sakazakii: Stress survival and virulence potential in an opportunistic foodborne pathogen. Gut Microbes.

[29] Bielik V., Hric I., Ugrayová S., Kubáňová L., Putala M., Grznár Ľ., Penesová A., Havranová A., Šardzíková S., Grendar M. (2022). Effect of High-intensity Training and Probiotics on Gut Microbiota Diversity in Competitive Swimmers: Randomized Controlled Trial. Sports Med. Open.

[30] Petersen L.M., Bautista E.J., Nguyen H., Hanson B.M., Chen L., Lek S.H., Sodergren E., Weinstock G.M. (2017). Community characteristics of the gut microbiomes of competitive cyclists. Microbiome.

[31] Sabater C., Iglesias-Gutiérrez E., Ruiz L., Margolles A. (2023). Next-generation sequencing of the athletic gut microbiota: A systematic review. Microbiome Res. Rep..

